# *Plasmodium falciparum Kelch-13* artemisinin partial resistance markers in Fort Portal, Western Uganda, 2024

**DOI:** 10.1128/aac.01755-24

**Published:** 2025-04-23

**Authors:** Welmoed van Loon, Emma Schallenberg, Emmanuel Mande, Patrick Musinguzi, Paul Ngobi, Sharon Atukunda, John Rubaihayo, Frank P. Mockenhaupt

**Affiliations:** 1 Institute of International Health, Charité Center for Global Health, Charité-Universitaetsmedizin Berlin 425077 , Berlin, Germany; 2 Infectious Diseases Institute, College of Health Sciences, Makerere University 242941, Kampala, Central Region, Uganda; 3 Fort Portal Regional Referral Hospital 279378, Fort Portal, Western Region, Uganda; 4 Department of Public Health, Mountains of the Moon University 187215, Fort Portal, Western Region, Uganda; The Children's Hospital of Philadelphia, Philadelphia, Pennsylvania, USA

**Keywords:** PfK13, PfMDR1, artemisinin resistance, Uganda, molecular markers of resistance

## Abstract

In Fort Portal, Uganda, artemisinin resistance-associated mutations in *Plasmodium falciparum Kelch13* (*n* = 126) were present in 4.8% (675V, 561H, and 441L). A mutation of unknown relevance, 490T, occurred in 9.5%. *PfMDR1* variants suggested increased lumefantrine tolerance (N86, 100%). Mutation 500N was absent, and 199S occurred in 12.8%. The latter is of unknown relevance. These data indicate an incipient emergence of artemisinin resistance in a crucial location between Rwandan and Ugandan resistance hotspots and hardly affected DR Congo.

## INTRODUCTION

Uganda, among the top three countries in malaria burden globally (1), is a hotspot of emerging partial artemisinin resistance (AR) of *Plasmodium falciparum* in East Africa (2). Malaria control relies on effective treatment with artemisinin combination therapy (ACT), composed of a fast-acting artemisinin derivative and a slower-acting partner drug, for example, lumefantrine or amodiaquine. Combination treatment is meant to prevent resistance. However, experience from Southeast Asia shows that once AR has emerged, resistance against the long half-life partner drugs can develop within years, resulting in clinical ACT treatment failure (3, 4). So far, this has not been observed in Africa. However, in Uganda, lumefantrine tolerance is increasing (5).

Molecular surveillance of resistance markers has a central role in the response to AR, helping identify hotspots and geospatial patterns. Certain mutations in the *P. falciparum Kelch-13* Propeller Domain (*PfK13*) associate with AR, referred to as validated or candidate *PfK13* markers. Tolerance to lumefantrine and amodiaquine is mediated by mutations in the *P. falciparum multi-drug resistance* 1 gene (*PfMDR1*), each drug exerting opposing selective effects (6–8).

**Fig 1:**
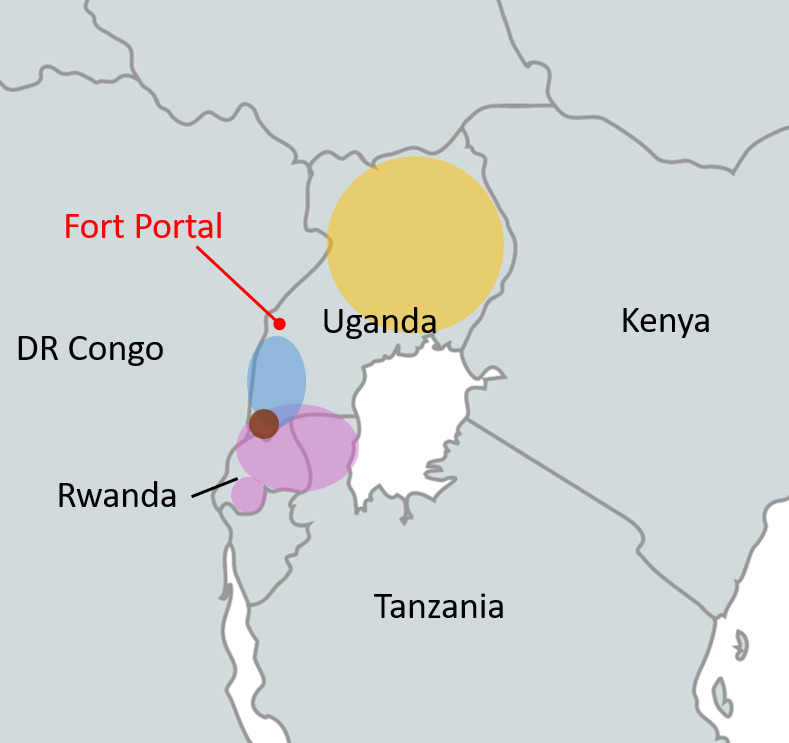
Occurrence of validated and associated *PfK13* markers of artemisinin partial resistance in East Africa, 2020–2023, color-coded by majority markers. Colored circles indicate regions where validated or candidate *PfK13* markers have been identified at prevalence >10% in 2020–2023. Yellow, majority of *PfK13* mutations found are 675V and/or 469Y (2); purple, majority *PfK13*-561H (2, 10, 12, 13); blue, majority *PfK13*-441L (2); and brown, majority *PfK13*-469F (2). Background map created with mapchart.net.

The aim of the present study was to contribute to unveiling the current situation of AR in a region of Uganda that has been little researched to date. The study area of Fort Portal, far western Uganda, is located between Ugandan and Rwandan AR hotspots (2, 9, 10), and so far hardly affected eastern DR Congo (10, 11). This area may potentially bridge *P. falciparum* populations with and without AR (Fig. 1). We genotyped *PfK13* and *PfMDR1* in samples from malaria patients presenting at health facilities in Fort Portal, from January to February 2024.

Malaria patients attending four health facilities in the area of Fort Portal located approximately 100 km from the Eastern DRC border with Uganda were invited to participate in this descriptive study from January to February 2024, following informed written consent. The study protocol was approved by the Mbarara University of Science and Technology Research Ethics Committee. Malaria was diagnosed by available rapid diagnostic tests for malaria at the health centers, and finger-prick blood samples were collected on Whatman 3 mm paper. DNA was extracted using a commercial kit (Qiagen, Hilden, Germany). A single PCR for *PfK13* spanning codons 426–709 (14) confirmed *P. falciparum* infection and generated amplicons for Sanger sequencing. Genotyping of *PfMDR1* region 1 and region 2, including markers at codons 86, 184, and 1,246, was done by PCR and sequencing (15) on a random selection of *P. falciparum*-positive samples. Sequences were aligned to reference sequences from laboratory strain 3D7 (PlasmoDB.org), using CodonCode Aligner. In addition, a novel putative marker for lumefantrine tolerance, *PfMDR1* Y500N (5), was typed by a high-resolution melting curve with a commercial SimpleProbe Assay (TIB MOLBiol, Berlin, Germany) on a Lightcycler 480 II (Roche). Positive and negative controls were included in all assays; mixed signals were counted as mutants.

Samples from 200 patients were analyzed, and 126 were confirmed by *PfK13* PCR to contain *P. falciparum*. Validated or candidate *PfK13* markers were present in 4.8% (6/126; 95% CI, 2.2%–10.0%). In addition, a *PfK13* mutation of so far unknown relevance, 490T, was observed in almost one in ten isolates (Table 1). One health center differed by mutation pattern compared with the others, as two validated markers appeared exclusively there (Table 2). No clear geographic distinction or patient population was identified for this center. For *PfMDR1*, 102 samples were assessed, wildtype allele N86 was present in all samples, and mutation 184Y slightly dominated over the wildtype allele. A *PfMDR1* mutation of unknown relevance, 199S, was present in more than 10%. The *PfMDR1* Y500N mutation was absent. In a random 47 samples sequenced for the *PfMDR1* region 2, wildtype allele D1246 prevalence was below 5% (Table 1).

**TABLE 1:** Prevalence of antimalarial drug-resistance markers in Fort Portal, Uganda, 2024

Gene	Mutation	No. reads	Prevalence mutant or mixed		Mixed
			(%, 95% CI)	(n)	(n)
*PfK13*	441L^*a*^	126	1.6 (0.4–5.6)	2	0
	490T		9.5 (5.5–15.9)	12	6
	561H^*a*^		1.6 (0.4–5.6)	2	0
	675V^*a*^		1.6 (0.4–5.6)	2	0
*PfMDR1* region 1	86Y	102	0	0	0
	184Y		62.8 (53.1–71.5)	64	13
	199S		12.8 (7.6–20.6)	13	2
	500N	125	0	0	0
*PfMDR1* region 2	1246Y	47	4.3 (0.5–14.5)	2	0
*PfMDR1* haplotype	N86-184F-D1246 (NFD)	43	60.5 (44.4–75.0)	26	N/A

^*a*^ Validated or candidate markers of artemisinin partial resistance.

**TABLE 2:** Prevalence of *P. falciparum Kelch*-13 markers by health centers, in Fort Portal, Uganda, 2024

*PfK13 m*utation	Fort Portal regional referral hospital (0° 39' 10.79" N, 30° 16' 33" E) (%, n/N)	Rukunyu Hospital (0° 15' 31" N, 30° 26' 58" E) (%, n/N)	Kibiito Health Centre IV (0°28'39.45" N, 30°11'43.1" E) (%, n/N)	Karugutu Health Centre IV (0°47'31.64" N, 30°13'36.7" E) (%, n/N)
WT	71.4, 15/21	90.9, 20/22	92.3, 36/39	84.1, 37/44
441L	4.8, 1/21	4.5, 1/22	0, 0/39	2.3, 1/44
490T	23.8, 5/21	4.5, 1/22	0, 0/39	13.6, 6/44
561H	0, 0/21	0, 0/22	2.6, 1/39	0, 0/44
675V	0, 0/21	0, 0/22	5.1, 2/39	0, 0/44

Molecular surveillance data for AR in Uganda are relatively abundant, but not for the far western Fort Portal area, located between AR hotspots and eastern DR Congo (Fig. 1). We found diverse AR-associated *PfK13* markers at a rather low combined prevalence of 4.8%. This contrasts with the situation in neighboring Rwanda and elsewhere in Uganda, where single AR markers reach 20%–40% (2, 10). *PfK13* 561H prevails in central Rwanda, 675V across Uganda (initially in the north), 441L in southern Uganda, and 469Y in northern Uganda (2). None of the markers dominates in Fort Portal, and 469Y is absent. As for lumefantrine, the *PfMDR1* pattern, particularly the fixation of the wildtype allele N86, suggests increased tolerance (6–8). *PfMDR1* 500N, recently suggested as a novel marker for lumefantrine tolerance, was absent (5). The *PfMDR1* mutation 199S (12.8%) is of unknown relevance, and it was previously seen at approximately 30% in northern Uganda in 2016/17, and at 2% in southern Rwanda in 2018/19 (16, 17). Its relevance needs to be evaluated *in vitro* or *ex vivo*. The same applies to a *PfK13* polymorphism of unclear significance, 490T, which we detected at almost 10%. The prevalence of these variants of unknown significance might indicate positive selection, although temporal follow-ups are required to confirm this. The molecular signatures in the Fort Portal area suggest that AR is starting to appear alongside reduced lumefantrine susceptibility.

AR is a multifactorial phenomenon, and the genetic background of *P. falciparum* plays a major part in the level of resistance and fitness (18, 19). Fort Portal appears to be a melting pot of various *P. falciparum* strains from several regions, where different AR mutations prevail at high prevalence. In these hotspot regions, mutated strains have been successfully selected for beneficiary factors, such as survival advantage under drug pressure and fitness. The overlapping geographical occurrence of these strains in the Fort Portal area may facilitate an accelerated reassembling of genetic factors favoring AR. Multi-drug-resistant *P. falciparum* in Southeast Asia likely developed through the recombination of strains with each their own drug resistance genotype (20). In Uganda, increased lumefantrine tolerance has already been observed (5). Particularly in regions like the study area with low *PfK13* mutation prevalence (bordering on so far hardly affected eastern DR Congo), the local selection of resistant parasite haplotypes should be rapidly monitored and targeted with scaled-up control measures. Facing a lack of alternatives to the ACTs and preventing the aggravation of AR in East Africa and its spread to other regions should be prioritized.

## Data Availability

The sequencing reads are available as raw ab1 files at doi.org/10.6084/m9.figshare.28704941.
